# Pancreaticojejunostomy Conducive to Biological Healing in Minimally Invasive Pancreaticoduodenectomy

**DOI:** 10.1007/s11605-022-05339-4

**Published:** 2022-05-11

**Authors:** Ying-Wen Gai, Huai-Tao Wang, Xiao-Dong Tan

**Affiliations:** Department of General Surgery, Shengjing Hospital of China Medical University, 36 Sanhao Street, Heping District, Shenyang City, 110004 Liaoning Province China

**Keywords:** Pancreaticojejunostomy, Pancreaticoduodenectomy, Pancreatic fistula, Minimally invasive surgery

## Abstract

**Background:**

Pancreaticojejunostomy, an independent risk factor for pancreatic fistula, is the cause of several postoperative complications of pancreaticoduodenectomy. As suturing in minimally invasive surgery is difficult to perform, more simplified methods are needed to guarantee a safe pancreatic anastomosis. The concept of “biological healing” proposed in recent years has changed the conventional understanding of the anastomosis, which recommends rich blood supply, low tension, and loose sutures in the reconstruction of the pancreatic outflow tract.

**Methods:**

A literature search was conducted in PubMed for articles on pancreaticojejunostomy published between January 2014 and December 2021. After following a due selection process, several techniques developed in accordance with the concept of biological healing that were found suitable for minimally invasive surgery and their related clinical outcomes were described in this review.

**Results:**

The incidence of clinically relevant pancreatic fistula associated with the presented techniques did not exceed 15.9%, indicating superior results compared to Cattell–Warren double-layer duct-to-mucosa anastomosis (incidence: approximately 20%). The features and drawbacks of these approaches have been enumerated from the viewpoint of biological healing.

**Conclusions:**

This review described several modified pancreaticojejunostomy techniques with the advantages of a simplified procedure and a lower incidence of pancreatic fistula. Surgeons can choose to apply them in clinical practice to improve patient prognosis.

## Introduction

Pancreaticoduodenectomy (PD) is a technically demanding surgery that involves multiple procedures to resect tumors in the pancreatic head and surrounding areas.^1,2^ Despite decades of technological advancement, the incidence of postoperative complications remains high, ranging from 30 to 50%.^3,4^ Postoperative pancreatic fistula (POPF), one of the most serious complications of PD, has a high incidence of 3 to 45%.^5,6^ Pancreatic juice exuded from the anastomotic stoma may corrode the stumps of the gastroduodenal arteries and other vessels, forming pseudoaneurysms with secondary hemorrhage, and leading to an increase in postoperative mortality.^7^ Studies have shown that the occurrence of POPF is related to several factors, such as the texture of the pancreas, blood supply to the tissues, the diameter of the main pancreatic duct (MPD), and pancreaticojejunostomy (PJ),^8^ among which PJ is an independent risk factor for POPF.^9^ Currently, there are more than 100 PJ techniques reported in the literature; nevertheless, none of them have been recognized as the gold-standard method to prevent pancreatic fistula.

Biological healing is a novel concept of PJ proposed by numerous surgeons in recent years.^10–13^ Regarding innovation, this theory emphasizes that excessive sutures are not conducive to the coalescence of anastomotic stoma and may even increase the risk of POPF. For example, if the needle distance is too near, the blood supply of the pancreatic remnant will be destroyed^10,11,14^; if there are too many sutures of the MPD, pancreatic juice seeping out of the pinholes may corrode the vascular stumps resulting in secondary hemorrhage.^11,15,16^ Furthermore, if the sutures are overtightened, the shear force of strings may lead to pancreatic edema and necrosis.^10,11,17^ Therefore, instead of pursuing mechanical tightness alone, we should consider factors such as the blood supply of the tissues, tension of the anastomotic stoma, healing of the pancreatic stump, and recovery of digestive function. “Wide, loose, and sparse” anastomosis has been recommended as a new goal for PJ.^10–12^

On the other hand, with the rapid development of enhanced recovery after surgery, minimally invasive surgeries have been widely performed in diverse areas. However, the PD procedure is relatively complex, with massive resection and reconstruction of the digestive tract. Particularly in PJ, the fulcrum effect of manipulation and poor hand–eye coordination further increases the difficulty in placing sutures via laparoscopy, which has reportedly hindered the development of minimally invasive pancreaticoduodenectomy (MIPD).^18,19^ Therefore, this study presents modified techniques that have been developed in accordance with the concept of biological healing, which simplifies the procedure and contributes to a lower incidence of postoperative complications. Surgeons can select preferred techniques and apply them to pancreatic anastomosis in MIPD.

## Materials and Methods

A literature search was conducted in PubMed using the following keywords: “pancreaticojejunostomy,” “pancreatic anastomosis,” and “pancreatic anastomotic technique.” This review focused on manuscripts published between January 2014 and December 2021. Selected articles on modified PJ techniques and their associated clinical outcomes are listed in Table 1. Suture materials and the advantages and disadvantages of each technique are summarized in Table 2.

**Table 1:** Clinical outcomes of the modified pancreaticojejunostomy techniques

Reference number, author	Publication year	Number of patients	CR-POPF rate (%)	Postoperative hemorrhage rate (%)	Delayed gastric emptying rate (%)	Bile leakage rate (%)	Abdominal infection rate (%)	Reoperation rate (%)	Operation time (min), mean ± SD/median (IQR)	Postoperative hospital stay (d), mean ± SD/median (IQR)	Mortality rate (%)
^24^ Zeng et al	2020	63	3.2%	1.6%	0%	6.3%	–	1.6%	352 ± 88	17.8 ± 8.1	3.2%
^13^ Wei et al	2018	104	7.7%	5.8%	35.6%	4.8%	4.8%	1.0%	240 ± 64	13 (11–18.5)	0%
^25^ Torres et al	2017	17	0	–	–	–	–	–	393 (310–590)	–	0%
^26^ Hong et al	2017	51	5.9%	2.0%	9.8%	9.8%	9.8%	2.0%	307 ± 69	16 ± 9	0%
^28^ Du et al	2019	31	6.5%	0%	3.2%	6.5%	6.5%	0%	321.8 ± 63.6	15.2 ± 4.6	0%
^30^ Liu et al	2021	89	6.7%	–	10.1%	4.5%	–	1.1%	253.7 ± 47.9	13.2 ± 9.6	1.1%
^31^ Liu et al	2018	81	0	–	9.8%	–	1.2%	0%	215 (180–310)	–	0%
^32^ Cai et al	2019	238	3.8%	1.3%	7.1%	2.5%		–	358 (220–495)	10.2 (5–19)	–
^34^ Ma et al	2022	71	7.0%	–	–	–	–	–	306 ± 60.7	12.9 ± 3.8	–
^35^ Ma et al	2021	62	15.9%	11.1%	41.3%	3.2%	1.6%	–	270 (225–335)	18 (15–22)	0%
^11^ Zhou et al	2021	149	9.40%	8.1%	7.4%	2.7%	10.1%	2.0%	260 ± 75	22.4 ± 13.81	0
^38^ Fujii et al	2014	120	2.5%	0%	2%	2%	6%	1%	436 ± 103	24 (12–60)	0%
^15^ Kobayashi et al	2021	222	3.2%	–	–	–	–	–	443 ± 6.6	–	0.9%
^39^ Li et al	2019	73	12.3%	4.2%	19.2%	0%	20.5%	2.7%	270 (2.16–8.05)	19 (7–65)	1.4%
^40^ Kojima et al	2018	101	3%	0%	2%	2%	9%	–	580 (520–626)	22 (11–90)	–
^16^ Menonna et al	2021	109	11.9%	8.2%	34.9%	–	–	11.9%	545 (460–605)	21 (14–30)	8.3%
^41^ Poves et al	2017	13	15.4%	7.7%	15.4%	–	–	0%	–	14 (7.5–15.5)	0%
^42^ Li et al	2018	188	4.3%	–		–	–	–	–	21.4 ± 11.2	
^44^ Ferencz et al	2020	130	6.9%	0.76%	0%	0%	–	5.3%	–	13 (7–75)	0.7%

Abbreviations: *CR-POPF*, clinically relevant postoperative pancreatic fistula; *IQR*, interquartile range; *SD*, standard deviation

**Table 2:** Suture material, needle type, and advantages and disadvantages of the modified pancreaticojejunostomy techniques

Types of PJ	Author	Suture material and needle type	Advantages	Disadvantages
Single-layer DTM interrupted PJ	^24^ Zeng et al	Transverse full-thickness sutures: 4–0 double-needles absorbable monofilament suture	Simplified procedure, uniformly distributed shear force, reduced damage of the tissues, alleviated tension in the anastomotic stoma, and enables the pancreatic juice to be drained out from the anastomotic stoma immediately	Not suitable for soft pancreas and thin MPD. Besides, if the MPD is not present at central area of the pancreatic stump, these methods are not applicable
	^13^ Wei et al	Transverse full-thickness sutures: 4–0 polyglactin 910 22 mm 1/2c (Vicryl) suture		
	^25^ Torres et al	Transverse full-thickness sutures: 5–0 double-needles polypropylene 17 mm 1/2c (Prolene) suture; circular running suture: 4–0 polypropylene 17 mm 1/2c (Prolene) suture	With the advantages mentioned above, the “clock-face” suturing is integrated to make the anastomosis more concise. Further, a circular running suture of the pancreatic capsule layer and the jejunal seromuscular layer is placed to reinforce the fixation	
Single-layer DTM continuous PJ	^26^ Hong et al	Single-stitch transpancreatic suture and the purse-string suture: 4–0 polydioxanone 17 mm 1/2c (PDS) suture; figure-of-eight continuous suture: 3–0 polydioxanone 17 mm 1/2c (PDS) suture	Simplified DTM anastomosis minimizes damage to the tissues, thus protecting its blood supply; insertion of the tube ensures a patent outflow of the pancreatic juice; and the procedure is greatly simplified, suitable for MIPD	Fixation of the drainage tube should be proper in case of displacement and the pancreatic juice may leak from the needle tract left by the transpancreatic suture on the MPD
	^28^ Du et al	“8-character” suture, purse-string suture and single-stitch transpancreatic suture: 5–0 polypropylene 17 mm 1/2c (Prolene) suture; circular running suture: 4–0 absorbable V-loc 180 barbed suture	Based on the advantages of Hong’s single-stitch PJ, the “8-character” suture further enhances the fixation of the drainage tube and the procedures of this technique are also quite simple	
	^30^ Liu et al	Support tube fixation: 5–0 polyglactin 910 13 mm 1/2c (Vicryl) sutures; figure-of-eight suture and single-layer full-thickness suture: 4–0 polypropylene 17 mm 1/2c (Prolene) sutures	DTM anastomosis is omitted by the insertion of a support tube, which protects the integrity of the MPD. Figure-of-eight sutures play a role in buttressing the pancreas and hemostasis. Further, the continuous full-thickness suture helps to simplify the procedure, provide uniform tension, and protect the blood supply of the tissues	Full-thickness continuous suture has the risk of injuring the MPD. Further, the tightening of the suture should be carried out slowly; otherwise, the shear force may damage the pancreas
	^31^ Liu et al	Circular continuous running suture: 5–0 double-needles polypropylene 17 mm 1/2c (Prolene) suture	The procedure is fairly simple and can be applied in cases with soft pancreatic texture and thin MPD. Furthermore, the DTM anastomosis is replaced by pancreatic stenting	Since the support tube is not fixed, there is a risk of displacement. Besides, the circular running sutures may leave dead cavity and increase the tension between the anastomotic stoma
Double-layer DTM continuous PJ	^32^ Cai et al	External layer circular running suture: 4–0 polypropylene 17 mm 1/2c (Prolene) suture; internal layer DTM anastomosis: 5–0 polydioxanone 13 mm 1/2c (PDS) suture	Continuous running suture is easy to perform with a well-distributed force, which can tighten the anastomotic stoma constantly without leaving a dead cavity. And the figure-of-eight suture at the posterior wall of the MPD also simplifies the procedure	Four-layer sutures inflict great damage to the tissues. Further, the application is limited by the pancreatic texture and MPD diameter
	^34^ Ma et al	External layer circular running suture: 4–0 absorbable V-loc 180 barbed suture; internal layer DTM anastomosis: 5–0 polydioxanone 13 mm 1/2c (PDS) sutures	Complete continuous running sutures are placed with the advantages of continuous suture mentioned above	
	^35^ Ma et al	The whole anastomosis: 4–0 polypropylene 17 mm 1/2c (Prolene) sutures	Continuous running sutures provide evenly distributed tension. Double purse-string sutures simplify the DTM anastomosis and reduce the risk of pancreatic leakage	When placing purse-string sutures, the MPD may get injured. Further, it should be confirmed that the tube is tightly fixed within the MPD in case of displacement. The tightening of the purse-string sutures should also be performed properly; otherwise, pancreatic juice may seep from the gap
	^11^ Zhou et al	The whole anastomosis: 4–0 polypropylene 17 mm 1/2c (Prolene) sutures	The combined use of the pancreatic stent and the purse-string sutures helps to ensure the patency of the pancreatic juice outflow and promote the healing of pancreaticointestinal epidermis along the tube	
Double-layer DTM interrupted PJ (modified Blumgart PJ)	^38^ Fujii et al	U-suture: 4–0 double-needles polypropylene 17 mm 1/2c (Prolene) sutures	Double-arm “mattress sutures” facilitate the wrapping of the anastomotic stoma by the jejunum and provide evenly distributed shear force	The procedures are still relatively complex for MIPD, and there is a risk of lateral injury of the MPD when placing the transpancreatic U-sutures
	^15^ Kobayashi et al	DTM anastomosis: 5–0 absorbable sutures; U-suture: 4–0 double-needles non-absorbable sutures	Reduced U-sutures are placed to minimize the risk of lateral injury	
	^39^ Li et al	U-suture and fixation of the pancreatic stent: 3–0 double-needles polypropylene 17 mm 1/2c (Prolene) sutures	DTM anastomosis is omitted by pancreatic stenting and posterior walls of the anastomotic stoma are superimposed on each other to eliminate the dead cavity	
	^40^ Kojima et al	U-suture: 4–0 double-needles polypropylene 17 mm 1/2c (Prolene) sutures	Peritoneal lavage is performed to clean the abdominal cavity and closed drains with dressing materials are placed to prevent retrograde infection	
	^16^ Menonna et al	U-suture: 3–0 double-needles polypropylene 17 mm 1/2c (Prolene) sutures	The number of the U-sutures are reduced to 2, and 2 half purse-string sutures are placed at both corners of the pancreas to strengthen the fixation	
	^41^ Poves et al	U-suture: 2–0 double-needles polypropylene 36 mm 1/2 c (Prolene) sutures; DTM anastomosis: 5–0 polyglactin 910 13 mm 1/2c (Vicryl) sutures	The distance and angle between the pancreas and the jejunum can be adjusted by external traction of the transpancreatic sutures out of the trocars; thus, suturing under a laparoscope is conveniently possible	
Modified invaginated PJ	^42^ Li et al	Purse-string suture: 3–0 polypropylene 17 mm 1/2c (Prolene) suture; pancreatic section suture: nylon thread sutures	The procedure is fairly simple. No “substantial sutures” of the pancreas are placed in the anastomosis and the pancreatic stump is completely embedded into the jejunum with reduced risk of pancreatic leakage. Additionally, invaginated PJ is suitable for soft pancreatic textures and thin MPD	Invaginated PJ is not applicable for a large pancreatic head. Further, the pancreatic stump will inevitably be corroded by the digestive fluids, leading to an increased risk of hemorrhage and exocrine dysfunction. Moreover, tension of the anastomotic stoma is relatively high, and tightening and fixation of the purse-string sutures must be performed appropriately
	^44^ Ferencz et al	Purse-string suture: 2–0 non-absorbable monofilament suture; U-shaped fixing sutures of the pancreas: 3–0 absorbable monofilament sutures	With advantages similar to those of Jiang’s PJ, this technique applies U-shaped fixing sutures to pull the pancreas into the jejunum, further reducing damage to the tissues	

Abbreviations: *MPD*, main pancreatic duct; *DTM*, duct-to-mucosa; *PJ*, pancreaticojejunostomy

## Results and Discussion

Cattell–Warren anastomosis (CWA) is a conventional and widely used duct-to-mucosa (DTM) PJ proposed by Cattell and Warren in 1956.^20^ By performing double-layer sutures, it forms a stable “mechanical connection” of the anastomotic stoma. However, since the method is relative complex and technically demanding, the incidence of POPF remains high according to the published studies (ranging from 17.8 to 22.9%).^16,21,22^ Biological healing of PJ is a novel concept which promotes fewer sutures and simpler procedures in the anastomosis. On the premise of proper fixation, fewer sutures help to maintain richer blood supply, lower tension, and inflict less damage to the tissues, thus enhancing the “psychological healing” of the pancreaticointestinal epidermis.^10–12^ Under this background, several PJ methods that are conducive to biological healing, which perform fewer sutures and apply modified suturing techniques, with a reduced incidence of POPF compared with CWA, have been presented as follows.

### Modified Duct-to-Mucosa Pancreaticojejunostomy

#### Single-Layer Interrupted Pancreaticojejunostomy

**Fig. 1:**
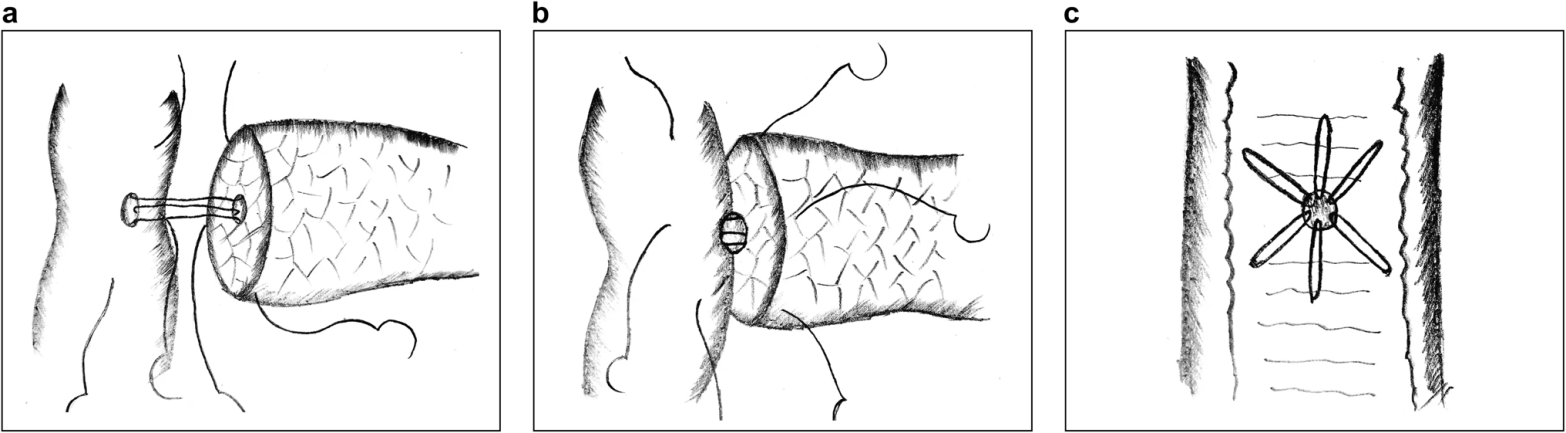
Six-stitch pancreaticojejunostomy. **a** The posterior wall of the anastomotic stoma is fixed with 3 full-thickness interrupted sutures at intervals of 60°. **b** The anterior wall is fixed in the same manner. **c** The interior view of the anastomosis from the intestinal lumen

CWA mainly applies double-layer sutures of the MPD with the jejunal mucosa layer, as well as the pancreatic capsule with the jejunal seromuscular layer. The procedure is complex and inflicts great damage to the pancreas. In 2015, Zhang et al. reported a single-layer anastomotic technique termed six-stitch PJ (SPJ).^23,24^ The procedural details are as follows:

1. Double needles are inserted from the inner side of the MPD and the jejunal mucosa. The full thickness of the pancreatic parenchyma is sutured with the jejunum at a distance similar to the radius of the pancreas, and the knots are placed outside (Fig. 1a).
2. The anterior wall is sutured at intervals of 60° in the same manner, accomplishing PJ in 6 stitches (Fig. 1b, c).

Unlike double-layer anastomosis, this technique regards the pancreas as a hollow organ. All 6 sutures are finished in one step, resulting in a simpler procedure and shorter operative time. The anastomotic layers in this technique are reduced, alleviating compression between the pancreaticointestinal tissues and abating cutting damage to the pancreatic stump caused by shear force. More importantly, in the CWA method, pancreatic juice extravasated from the pancreas might be stored between the double layers and accumulate into major leaks. By contrast, single-layer PJ enables the exudates to be drained immediately into the abdominal cavity, thus lowering the occurrence of POPF.^13^ However, disadvantages are that this technique is not suitable for soft pancreatic texture and thin MPD. Besides, if the MPD does not locate at central area of the pancreatic stump, the suturing of the tissues will not be symmetric, which may influences the stability of the anastomotic stoma.

Torres et al. reported the modified Heidelberg technique in 2017.^25^ Compared with SPJ, this technique integrates the concept of “clock-face” anastomosis, which regards the MPD as a clock and inserts 6 transpancreatic sutures at the 2, 4, 6, 8, 10, and 12 o’clock positions. Additionally, circular running sutures between the pancreatic parenchyma and the jejunum seromuscular layer are added for further fixation.

Wei et al. reported a method almost identical to SPJ, termed “modified 1-layer DTM pancreaticojejunostomy” in a retrospective study in 2018, which compared this technique with double-layer DTM anastomosis.^13^ The final results showed that the incidence of POPF was lower in the one-layer group.

#### Hong’s Single-Stitch Duct-to-Mucosa Pancreaticojejunostomy

Hong’s single-stitch PJ is a DTM PJ technique reported by Hong et al. in 2017 that is based on T-tube drainage in biliary surgery.^26^

**Fig. 2:**
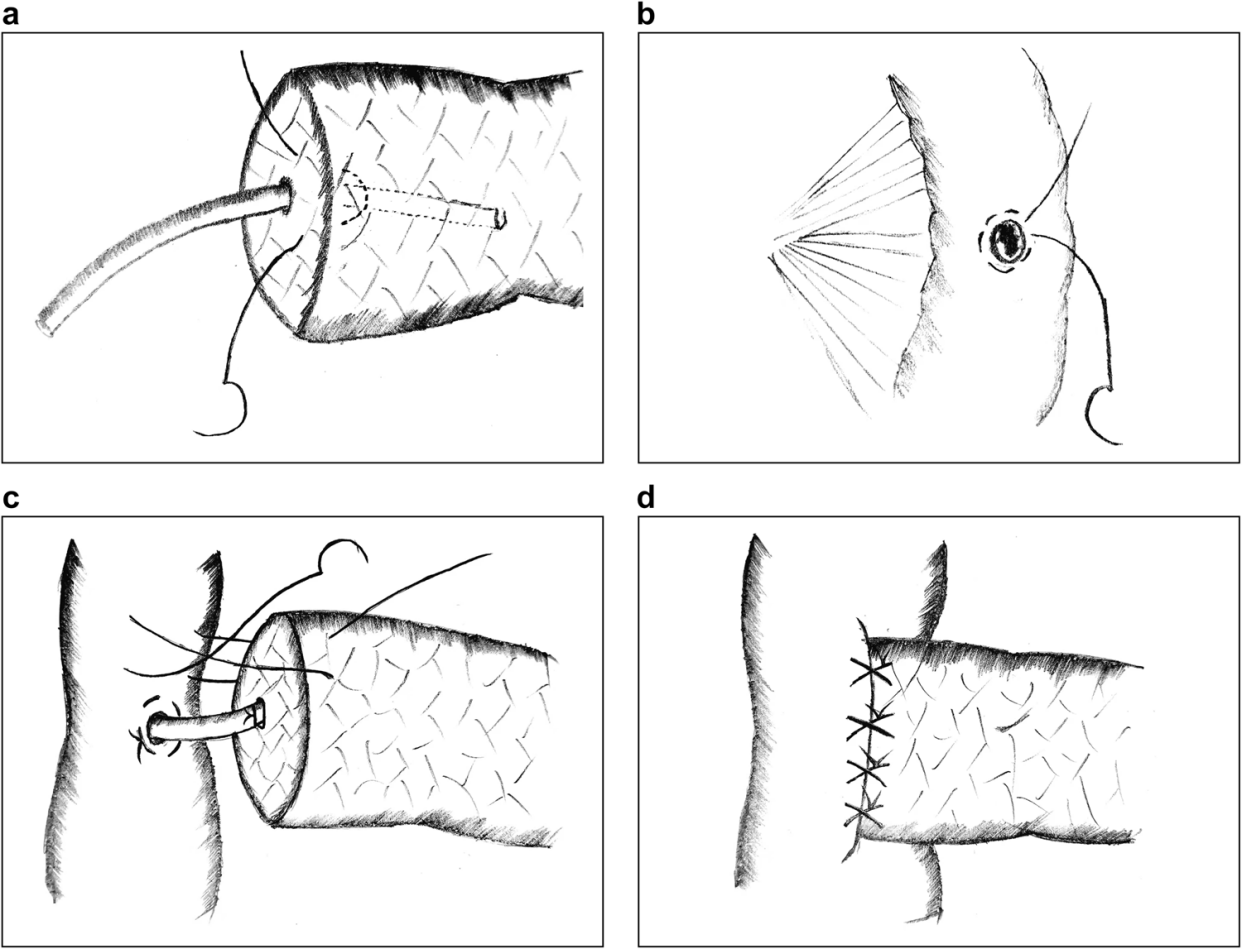
Hong’s single-stitch pancreaticojejunostomy. **a** A full-thickness penetrating suture is placed to fix the tube. **b** A purse-string suture is made around the small hole in the jejunum. **c**, **d** Several figure-of-eight continuous full-layer sutures are placed to join the pancreas with the jejunum

1. The MPD and the drainage tube are penetrated with a single suture through the full thickness of the pancreas. Subsequently, a knot is tied around the tube for fixation (Fig. 2a).
2. A corresponding hole is made in the jejunum, and a purse-string suture is placed around the hole to fix the tube at the jejunal side (Fig. 2b).
3. Several figure-of-eight continuous full-layer sutures are placed to anastomose the pancreatic parenchyma with the jejunal seromuscular layer (Fig. 2c).^27^

The features of this method are that it places a single-stitch penetrating suture to fix the drainage tube with the pancreas and that it uses the tube to divert pancreatic juice instead of placing DTM sutures. The mechanism of Hong’s PJ is similar to bridge catheter internal drainage, which reconstructs the pancreatic outflow tract through the healing of the pancreaticointestinal epithelium along the tube. Advantages of this technique include (1) the use of fewer sutures in the PJ minimizes damage to the tissues protecting the blood supply of the pancreatic stump; (2) the insertion of the tube ensures the patency of pancreatic juice outflow, thus preventing pancreatic leakage at the anastomotic stoma; and (3) the procedure is greatly simplified, making it especially suited for MIPD.

However, a disadvantage is that the penetrating suture inevitably destroys the integrity of the MPD. As a result, pancreatic juice may seep through the pinholes and corrode the vascular stumps. Furthermore, surgeons must ensure proper fixation of the tube during the operation; its displacement may lead to a severe pancreatic fistula.

**Fig. 3:**
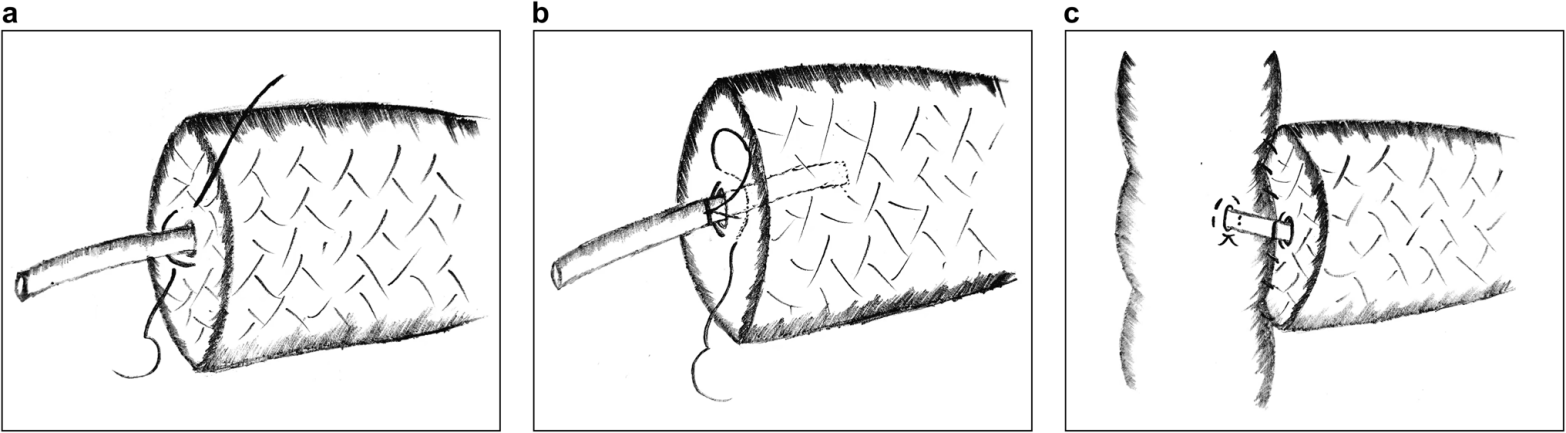
An “8-character” suture pancreaticojejunostomy. **a** An “8-character” suture is made around the main pancreatic duct. **b**–**c** A penetrating suture and a purse-string suture are placed to fix the pancreatic stent with the digestive tract at both sides

Du et al. explored the modified “8-character” suture method based on Hong’s PJ in 2019.^28^ In this technique, an “8-character” suture centered at the pancreatic stent is placed to maintain adhesion of the MPD with the tube in case of pancreatic leakage from the gap (Figure 3a). To ensure an enhanced fixation of the pancreatic stent, a penetrating suture similar to that in Hong’s PJ and a purse-string suture around the hole on the jejunum are placed on both sides (Figure 3b, c). Finally, a circular running suture is made in the pancreatic parenchyma and jejunum seromuscular layer to accomplish external layer anastomosis.

#### Single-Layer Continuous Suture Pancreaticojejunostomy

**Fig. 4:**
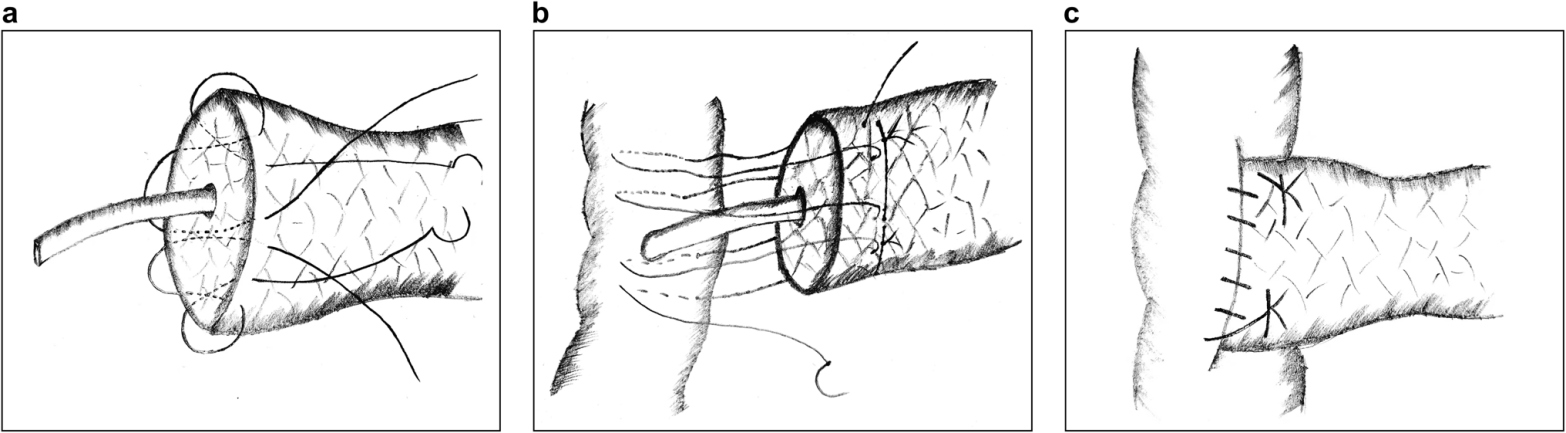
Single-layer continuous suture pancreaticojejunostomy. **a** Two horizontal figure-of-eight traversing sutures are placed to buttress the pancreas. **b**, **c** A single-layer continuous full-thickness suture is placed to join the pancreas with the jejunum

In 2018, Liu et al. reported the single-layer continuous suture (SCS) PJ technique for robotic pancreaticoduodenectomy.^29^ The main steps of the procedure are as follows:

1. Two horizontal figure-of-eight traversing sutures are placed to buttress the pancreas (Fig. 4a).
2. The full thickness of the pancreas is continuously sutured with the jejunal seromuscular layer from back to front, with a total of 4–6 stitches (Fig. 4b).
3. The suture is slowly tightened and tied with the spare thread of the figure-of-eight sutures to secure the anastomosis (Fig. 4c).

Robotic surgery lacks force feedback. Therefore, it is difficult to precisely control the force of intermittent sutures; both loose and overtightened sutures may lead to rupture or necrosis of the anastomotic stoma. Hence, SCS PJ applies continuous full-thickness sutures, which simplifies the procedure in robotic surgery and provides evenly distributed tension between the pancreas and jejunum. Furthermore, the insertion of the support tube for internal drainage replaces the DTM anastomosis and protects the integrity of the MPD without pinholes. Two figure-of-eight sutures also prevent bleeding and pancreatic fistula from the pancreatic stump.^30^ However, it should be noted that when placing full-thickness sutures, we should be careful not to accidentally injure the MPD. Moreover, tightening the sutures should be done slowly and cautiously; otherwise, the shear force might result in cutting damage and postoperative hemorrhage.

In the same year, Liu et al. presented another version of SCS PJ, which places a circular running suture around the pancreatic parenchyma and the jejunal seromuscular layer.^31^ The DTM anastomosis is omitted by the support tube without fixation. The main advantage of this technique is that it is a greatly simplified procedure, regardless of pancreatic texture and MPD size. According to current data, on an average it takes only 7 min to accomplish this technique. However, circular running sutures may result in greater tension between the pancreas and jejunum than full-thickness sutures, which could lead to avulsion of the tissues. In addition, the sutures must be tight enough to fix the pancreatic remnants and eliminate the dead cavity. Furthermore, there is a risk of tube displacement.

#### Double-Layer Continuous Suture Pancreaticojejunostomy

**Fig. 5:**
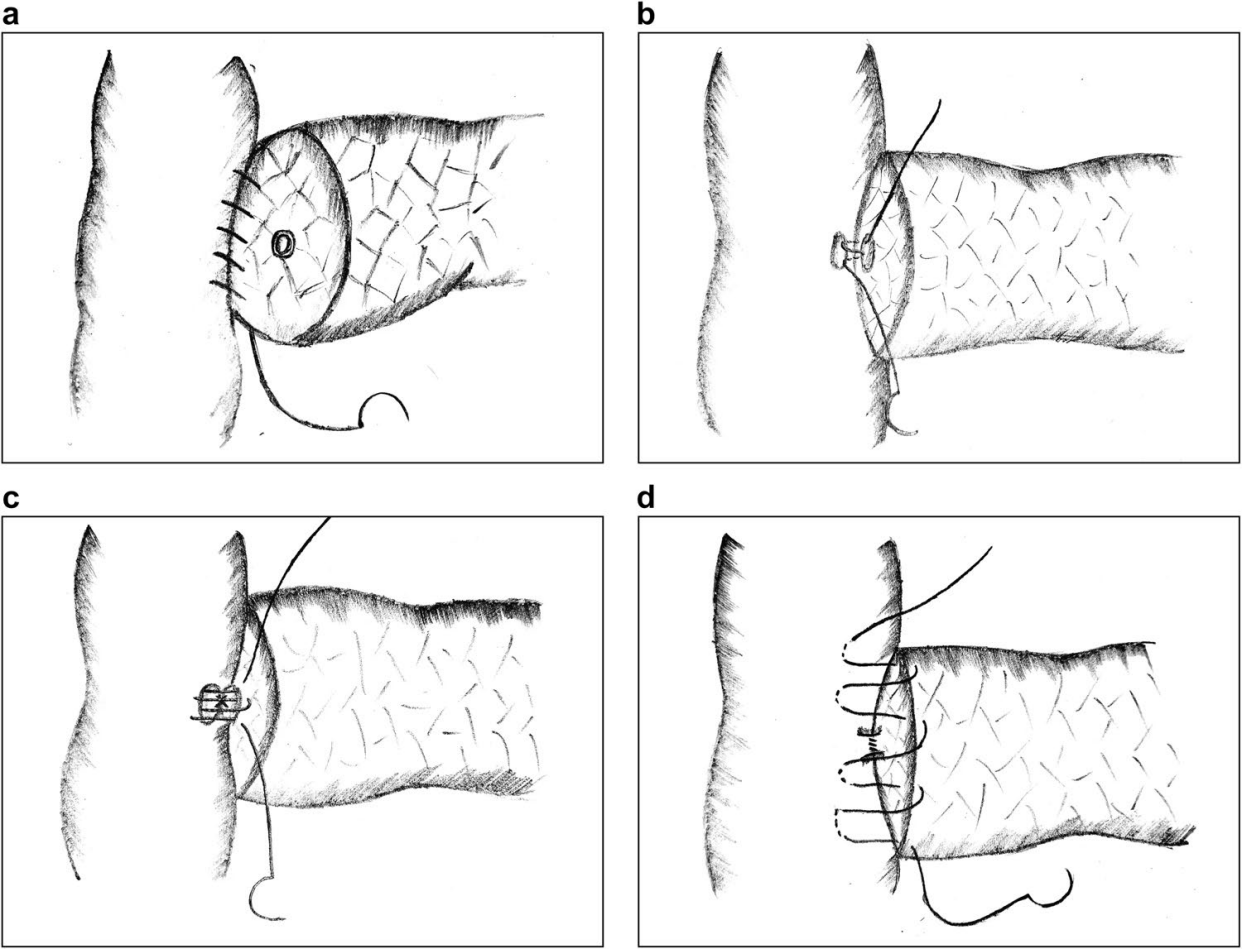
Bing’s pancreaticojejunostomy. **a** A running suture to fix the posterior wall of the external layer. **b** A figure-of-eight suture to fix the posterior wall of the inner layer. **c** Another running suture to fix the anterior wall of the inner layer. **d** The first running suture is continuously sutured to fix the anterior wall of the external layer

In 2018, Cai et al. proposed Bing’s PJ.^32^ The main procedure includes the following:

1. A continuous running suture joining the pancreatic stump with the jejunal seromuscular layer is placed without tightening (Fig. 5a).
2. A plastic catheter of matched size is inserted into the MPD, and a figure-of-eight suture is placed to fix the posterior wall of the MPD with the jejunum (Fig. 5b).
3. A running suture is placed on the anterior wall of the MPD with the jejunum (Fig. 5c).
4. Suturing circularly between the pancreatic stump and the jejunal seromuscular layer is continued until the anastomotic stoma is completely closed (Fig. 5d).

Since intermittent sutures are time-consuming and technically demanding via laparoscopy, Bing’s PJ is advantageous as it applies multi-layer continuous running sutures in the anastomosis, which are easy to perform with a well-distributed force. Moreover, continuous sutures can tighten the anastomotic stoma constantly without leaving a dead cavity, thereby preventing the retention and extravasation of pancreatic juice.^33^ Because it is difficult to place sutures on the posterior wall of the MPD, the figure-of-eight suture simplifies the procedure. A disadvantage is that four-layer sutures inflict great compression and damage to the pancreaticointestinal tissues. Furthermore, the application of this technique is limited by the size of the MPD and the texture of the pancreas.

Ma et al. presented a modified laparoscopic DTM anastomotic technique similar to Bing’s PJ in 2021.^34^ However, this technique involves complete double-layer running sutures instead of a figure-of-eight suture on the posterior wall. A retrospective study conducted by this team showed that this technique had satisfactory clinical outcomes.

#### Modified Double Purse-String Continuous Suture Pancreaticojejunostomy

**Fig. 6:**
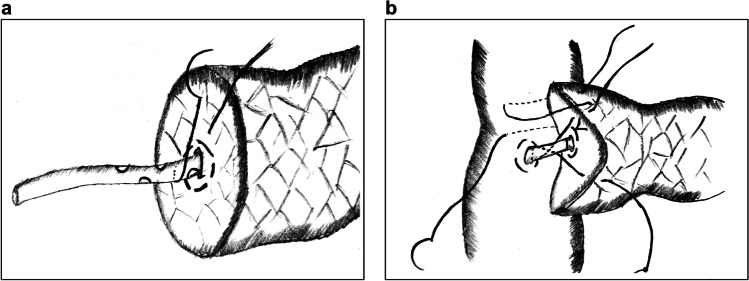
Modified double purse-string continuous suture pancreaticojejunostomy. **a** A purse-string suture around the main pancreatic duct to fix the tube at the pancreas. **b** A second purse-string suture for simplified duct-to-mucosa anastomosis

Double purse-string continuous suture PJ is a novel technique reported by Ma et al. in 2021.^35^ The main procedure is as follows:

1. A support tube with several holes on the surface is inserted into the MPD.
2. A purse-string suture is placed around the MPD to fix the tube at the pancreas (Fig. 6a).
3. A circular continuous suture is placed to fix the posterior wall of the pancreatic parenchyma and the jejunum seromuscular layer.
4. A suture is inserted from the anterior wall of the pancreas and pierced out at the pancreatic section behind the support tube. Afterwards, the suture is used to perform a purse-string suture around the incision in the jejunum without knotting. The suture is then reversed from the back of the tube and taken out through the anterior wall of the pancreas. Finally, the suture is knotted on the pancreas. Thus, a cross fixation similar to a figure-of-eight suture is made to complete the simplified DTM anastomosis (Fig. 6b).
5. The anterior wall of the pancreas and the jejunum are sutured to close the external layer of the anastomotic stoma.

The purse-string suture in PJ has become a common anastomotic method to simplify DTM sutures, lowering the difficulty of the procedure and inflicting little damage to the pancreas. With this technique, double purse-string sutures further strengthen the fixation of the inner anastomosis, thereby reducing the incidence of POPF. However, when placing purse-string sutures, special attention should be paid to avoid damaging the MPD, and the tube should be tightly fixed to the pancreas in case of displacement. Although holes on the catheter prevent obstruction of the outflow tract, pancreatic juice may also leak into the abdominal cavity and cause POPF.

**Fig. 7:**
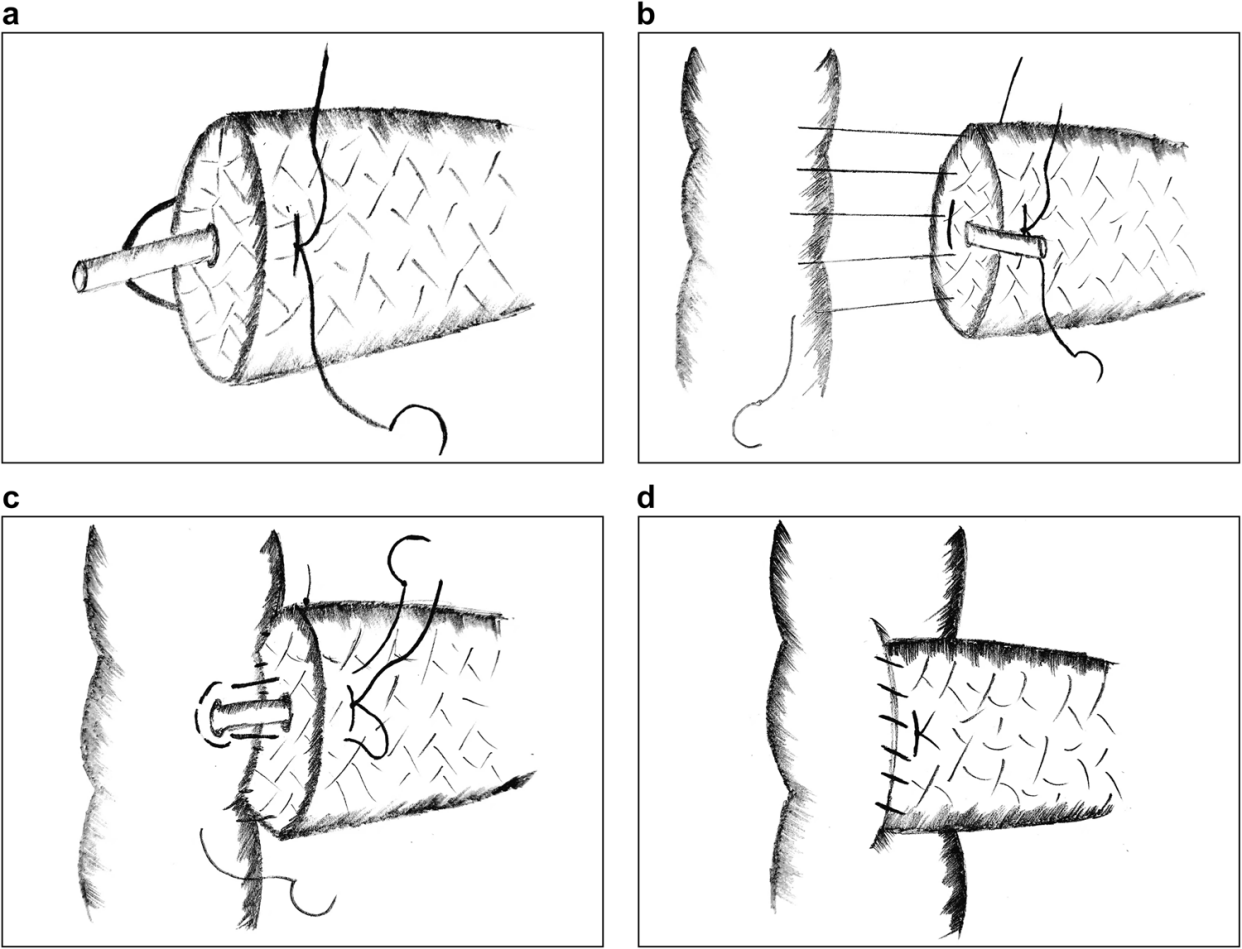
Three sutures PJ. **a** A U-suture around the MPD with preserved needles. **b** A circular continuous suture of the pancreatic parenchyma and the jejunum seromuscular layer at the posterior wall. **c** A 270° purse-string suture around the hole on the jejunum to fix the pancreatic stent. **d** Another continuous running suture to fix the anterior wall

In 2021, Zhou et al. reported the “three sutures” PJ, which also applies purse-string suture in the anastomosis.^11^ First, a U-shaped suture penetrating the pancreas is placed around the MPD, and the needles are preserved for further use after knotting (Fig. 7a). Then, circular continuous suturing of the pancreatic parenchyma and the jejunum seromuscular layer is performed to fix the posterior wall without knotting (Fig. 7b). Subsequently, a corresponding hole is made in the jejunum and a U-suture is used to make a 270° purse-string suture around the hole, which plays a role in pancreatic stent fixation (Fig. 7c). Finally, the anterior wall of the external layer is continuously sutured to close the anastomotic stoma (Fig. 7d).

### Modified Blumgart Pancreaticojejunostomy

As a classic end-to-side duct-to-mucosa (DTM) technique, Blumgart PJ has no restrictions on the texture of the pancreas or the size of the MPD.^36^ Longitudinal U-sutures through the pancreas can minimize shear force and prevent laceration.^37^ However, due to its relatively complex procedure, modifications to this technique are still needed for wider applications in MIPD.

In 2014, Fujii et al. reported a modified Blumgart PJ technique with the following improvements:^38^

**Fig. 8:**
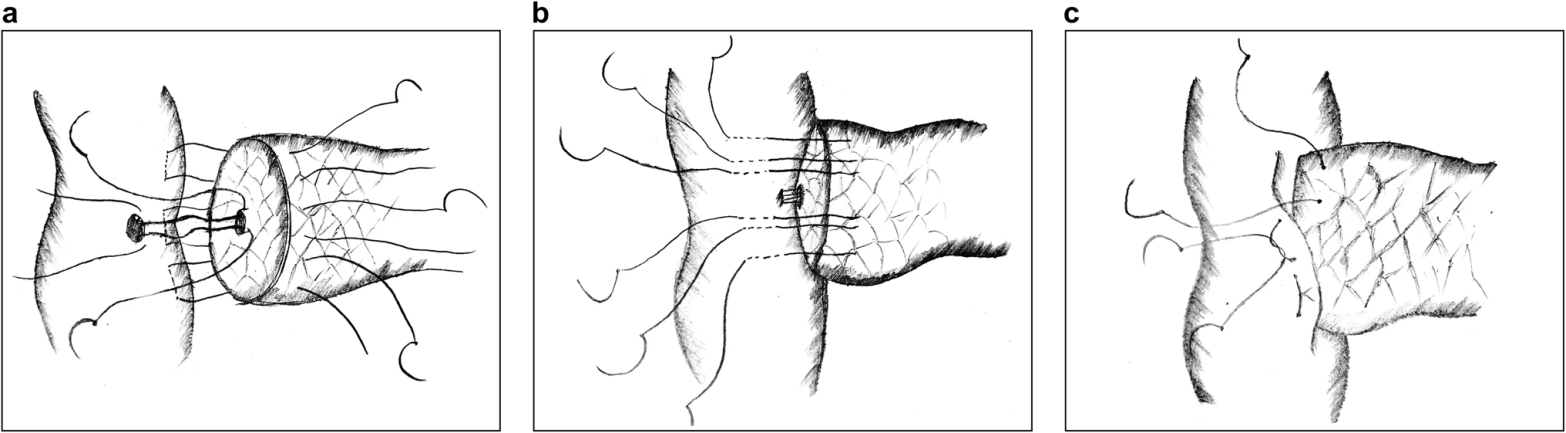
Modified Blumgart pancreaticojejunostomy. **a** U-sutures of the pancreatic stump with the seromuscular layer of the jejunum. **b** After finishing the duct-to-mucosa anastomosis, the jejunum is continuously anastomosed with the U-sutures at the anterior wall. **c** Knots are placed on the jejunum

1. The number of U-sutures between the pancreas and jejunum is reduced from 4–6 to 1–3, which simplifies the procedure (Fig. 8a).
2. The original Blumgart PJ method places penetrating U-sutures through the parenchyma that are knotted on the pancreas; however, the modified method does not knot after suturing the pancreas. Instead, it continues to anastomose the anterior wall of the jejunal seromuscular layer with both ends of the needle (Fig. 8b). Finally, the knots are tied on the jejunum, completing the embedded “double-arm” mattress sutures (Fig. 8c).

The feature of this modified Blumgart PJ is the U-shaped “double-arm” mattress sutures, which facilitate the wrapping of the pancreatic stump by the jejunum, thereby lowering the risk of pancreatic juice extravasation. Additionally, since the strings are knotted at the jejunum, the shear force on the anterior and posterior walls is uniform, further reducing the cutting damage to the pancreas.

There are several other techniques similar to Fujii et al.’s PJ. Kobayashi et al. reported the COMPAS anastomosis in 2021.^15^ The main difference is that the number of U-sutures is reduced to 2. They proposed that 2 sutures can provide enough tension to fix the PJ and that the reduced number of sutures lowers the risk of lateral injury. Li et al. reported another modified technique with the following improvements: first, the DTM anastomosis is replaced by an internal pancreatic duct stent without direct sutures, and second, the back-wall sutures are superimposed on each other to eliminate the gap between the posterior wall, further reducing the risk of pancreatic leakage.^39^ Kojima et al. reported a nearly identical method to Fujii et al.’s PJ termed the modified Blumgart anastomosis with the “complete packing method,” which includes peritoneal lavage and wound dressing to reduce the incidence of POPF.^40^ Menonna et al. presented a novel version of the Blumgart technique with additional modifications, which adds 2 half purse-string sutures at the corner of the pancreas to attach the pancreatic stump to the jejunum. They also agreed that 2 U-sutures are sufficient to fix the anastomotic stoma and minimize damage to the pancreaticointestinal tissues.^16^

**Fig. 9:**
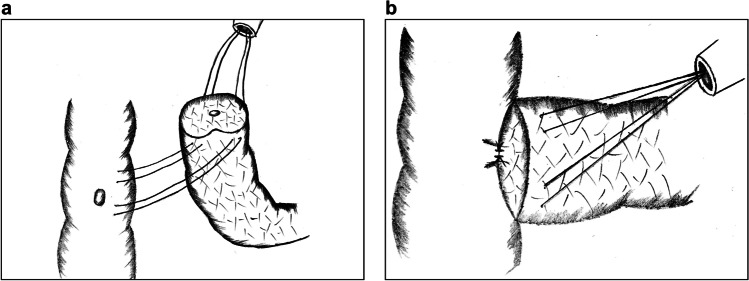
Laparoscopic Blumgart pancreaticojejunostomy. **a** The sutures are loosened to increase the space for manipulation. **b** The sutures are tightened to close the gap between the pancreas and the jejunum

Poves et al. proposed a laparoscopic Blumgart PJ in 2017.^41^ A feature of this technique is that after making U-sutures in the posterior wall, the suture ends are taken out of the trocars without knotting, and surgeons can subsequently adjust the tensions of the sutures to control the distance and angle between the pancreas and jejunum. If the sutures are loosened, there will be more space to place the DTM sutures (Figure 9a). If the sutures are tightened, the pancreas will be drawn toward the jejunum, which makes it easier for surgeons to fix the knots, thus increasing convenience under laparoscopy (Figure 9b).

### Modified Invaginated Pancreaticojejunostomy

**Fig. 10:**
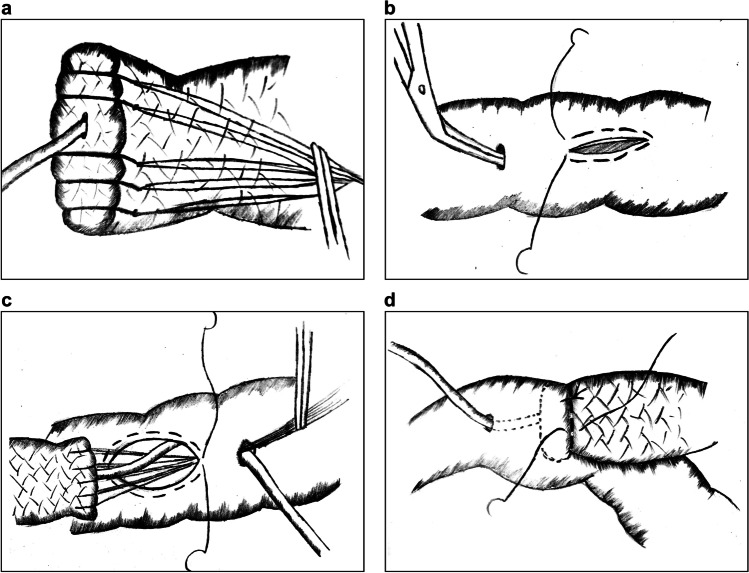
Jiang’s pancreaticojejunostomy. **a** The pancreatic stump is sutured intermittently with several nylon sutures. **b** An incision corresponding to the pancreatic stump is made in the jejunum, and a purse-string suture is placed nearby. **c** The pancreas is placed in the jejunum by pulling the nylon sutures from a small hole. **d** The purse-string suture is tightened, and 2–3 relaxation sutures are placed to fix the anastomotic stoma

In 2018, Li et al. reported a modified invaginated PJ technique termed Jiang’s PJ with further improvements.^42^ The steps involved are as follows:

1. The pancreatic stump is sutured with several intermittent nylon sutures to prevent hemorrhage (Fig. 10a).
2. An incision slightly smaller than the pancreatic stump is made on the jejunum. A purse-string suture is placed nearby without tightening (Fig. 10b).
3. The pancreas is implanted into the jejunum through a small hole beside the incision (Fig. 10c), and the purse-string suture is tightened to close the anastomotic stoma. The pancreatic capsules and jejunal seromuscular layer are further fixed with 2–3 relaxation sutures (Fig. 10d).

Regarding modifications, this invaginated PJ technique does not extend any “substantial sutures” to the pancreatic parenchyma, thus preventing tissue damage. The pancreatic stump is completely embedded into the jejunum, further reducing the risk of pancreatic juice extravasation from small pancreatic ducts and needle tracts. Moreover, this technique is not limited by the texture of the pancreas or the MPD diameter, and the procedure is fairly simple. Reports have shown that it takes only 5–7 min to complete Jiang’s PJ via laparoscopy, which is suitable for minimally invasive surgeries.

However, there are also some drawbacks. First, if the pancreatic head is too large or if it is difficult to free the pancreas from the surrounding tissues, this method cannot be applied. Second, intestinal fluid and pancreatic juice inevitably corrode the pancreatic stump embedded in the jejunum, which is prone to postoperative hemorrhage and exocrine dysfunction in the long term.^43^ Third, tightening the purse-string suture appropriately is the focus of this PJ technique, since forceful pulling may impair the blood supply of the anastomotic stoma, leading to delayed healing or necrosis, while loose fixation may result in separation of the pancreatic stump from the jejunum, causing serious complications.

Ferencz et al. developed another modified invaginated method similar to Jiang’s PJ in 2020.^44^ This technique places 2 U-sutures at the upper and lower edges of the pancreas with the jejunum rather than interrupted pancreatic section sutures as seen in Jiang’s PJ. In addition, the pancreatic stump is implanted into the bowel lumen by pulling the 2 U-sutures. Since the procedure is a simplified version, it is a recommended invagination technique with little damage to the pancreaticointestinal tissues in MIPD.

## Conclusion

This paper introduces several modified PJ techniques, which were conducive to biological healing and simplified in procedures. The incidence of POPF and related complications with these techniques was low, thus improving patient prognosis. Surgeons can choose to apply them for pancreatic reconstruction in MIPD.
